# Association of dietary patterns and sarcopenia in the elderly population: a cross-sectional study

**DOI:** 10.3389/fragi.2023.1239945

**Published:** 2023-08-25

**Authors:** Boshi Wang, Yanan Wei, Lin Shao, Menghan Li, Xue Zhang, Wei Li, Shilong Zhao, Xin Xia, Peng Liu

**Affiliations:** ^1^ Department of Clinical Nutrition, Peking University People’s Hospital, Beijing, China; ^2^ Department of Geriatrics, Peking University People’s Hospital, Beijing, China; ^3^ National Clinical Research Center for Geriatric Diseases, West China Hospital, Sichuan University, Chengdu, China; ^4^ Geriatric Healthcare and Medical Research Center, Sichuan University, Chengdu, China

**Keywords:** sarcopenia, prevalence, dietary patterns, older adults, cross-sectional study

## Abstract

**Background:** Sarcopenia, defined as the loss of muscle mass and strength, has been associated with increased hospitalization and mortality. Dietary pattern analysis is a whole diet approach which in this study was used to investigate the relationship between diet and sarcopenia. This study aims to estimate the prevalence of sarcopenia and explore possible factors associated with it among a large population in Beijing, China.

**Methods:** A cross-sectional study with 1,059 participants aged more than 50 years was performed. Sarcopenia was defined based on the guidelines of the Asian Working Group for Sarcopenia. The total score of the MNA-SF questionnaire was used to analyse nutrition status. The baseline demographic information**,** diet structure and eating habits were collected by clinicians trained in questionnaire data collection and anthropometric and bioimpedance measurements.

**Results:** The overall prevalence of sarcopenia was 8.8% and increased with age: 5%, 5.8%, 10.3% and 26.2% in the 50–59, 60–69, 70–79, and ≥80 years groups, respectively. Marital status (with or without a spouse) was not an independent factor associated with sarcopenia adjusted by age and sex. However, nutritional risk or malnutrition, vegetable diet, advanced age and spicy eating habits were risk factors for sarcopenia. Meanwhile, daily fruit, dairy and nut consumption were protective factors against sarcopenia adjusted by age, sex, income status and spouse status.

**Conclusion:** Although further studies are required to explore the association between healthy dietary patterns and the risk of sarcopenia, the present study provides basic data for identifying correlates of sarcopenia in elderly Chinese individual.

## Introduction

Sarcopenia is an age-dependent syndrome characterized by a progressive loss of muscle mass combined with reduced muscle strength and/or physical performance (Chen et al., 2014). It is now recognized that the decrease in skeletal muscle mass and muscle strength starts at ∼40 years, leading to sarcopenia appearing earlier in life (Cruz-Jentoft and Sayer, 2019). In Asia, the prevalence of sarcopenia according to the Asian Working Group of Sarcopenia (AWGS) 2014 definition was estimated at 4.1%–11.5% in the general older population (Beaudart et al., 2014; Chen et al., 2016). Due to the escalation of the reported prevalence in elderly populations, sarcopenia leads to a worse quality of life and higher social burden, as well as healthcare costs (Beaudart et al., 2014; Xu et al., 2022).

The etiology and underlying mechanisms of sarcopenia are complicated and multifactorial, reportedly involving malnutrition, reduced exercise, immune imbalance, neuromuscular junction degeneration and oxidative stress (de Oliveira Neto et al., 2021; Kamper et al., 2021). Dietary interventions, such as protein, vitamin D or antioxidant supplements, may prevent or at least delay the onset of sarcopenia by improving some of these pathological processes (Chew et al., 2021; Choi et al., 2021). However, compared with the monitoring of single nutrients, dietary patterns focus on regional characteristics, the integrity of dietary structures, the interaction of various nutrients and the synergy between foods and nutrients. Thus, this type of study may be more effective in examining the dietary influence on sarcopenia and may facilitate translation of findings into locally appropriate public health recommendations (Reedy et al., 2017). According to a systematic review, adhering to healthy dietary patterns may maintain gait speed in older adults. However, the evidence base is limited by the risk of publication bias (Van Elswyk et al., 2022). More high-quality research is needed to reveal the relationship between healthy dietary patterns and sarcopenia. Therefore, we conducted a community-based cross-sectional study to explore the association between dietary patterns and sarcopenia in a large-scale elderly population in northern China.

## Methods

### Study design

The current research is a cross-sectional analysis that finished data collection in May 2022. The study was approved by the Ethical Review Committee of Peking University People`s Hospital with the committee’s reference number 2021PHB119-001. The method of sampling was cluster sampling, and three communities were randomly selected for investigation. All participants aged 50 or older were enrolled and provided signed informed consent. This research is supported by Grant No. 2020YFC2005600/05 from the National Key R&D Program of China.

### Participants

The study included participants aged 50 and above from the three communities and excluded participants who 1) refused to sign the informed consent; 2) suffered from terminal disease; 3) had a major disability or mental illness; and 4) were unable to cooperate with investigators.

### Data collection

All data collectors are clinicians trained in questionnaire data collection and anthropometric and bioimpedance measurements. The baseline demographic information included the following: 1) General personal data: age, gender, spouse status and income status; 2) diet structure and eating habits: balanced diet, eating habits (light, salty, sweet and spicy), dietary intake (fruits, vegetable, meat, fish, eggs, pickle, carbohydrate, garlic, dairy, nut, thallophyte and vitamin). Anthropometric measurements included height and weight.

### Nutritional status

The Mini Nutritional Assessment short-form (MNA-SF) was used to assess the nutrition status of elderly individuals. The MNA-SF is sensitive, specific, and accurate in identifying nutrition risk (Kaiser et al., 2009). The total score on the scale is 14. The higher the score is, the better the nutritional status. In addition, 0–7 points indicated malnutrition, 8–11 points indicated a risk of malnutrition, and 12–14 points indicated normal nutritional status.

### Dietary patterns assessment

The dietary survey method adopted in this study is the simplified version of the food frequency inquiry method (FFQ), which involves inquiring about the frequency of food consumption in 12 kinds of food: fruits, vegetables, meat, fish and other aquatic products, eggs, soy products, salted vegetables, white sugar or fructose, garlic, dairy products, nuts, bacteria and algae. In addition, it is also investigated whether the dietary habits are balanced between meat and vegetables, primarily meat-based or vegetarian; Types and intake of staple foods; Dietary taste (light/salty/sweet/prefers spicy food/prefers cold food/no above habits).

#### Sarcopenia assessment

Sarcopenia was measured by the diagnostic criteria of the AWGS 2019, which is widely used in the diagnosis of sarcopenia in Asia, considering the loss in muscle mass, muscle strength and physical performance (Chen et al., 2020). According to the AWGS, appendicular muscle mass (male: < 7.0 kg/m^2^, female: < 5.7 kg/m^2^) for bioelectrical impedance analysis (BIA) is considered a loss of muscle mass (Chen et al., 2020). The AWGS also suggests that handgrip strength of < 28 kg and < 18 kg for men and women is defined as decreased muscle strength (Chen et al., 2020). The 6 m walking test < 1.0 m/s and Short Physical Performance Battery (SPPB) ≤ 9 are recommended for the evaluation of physical ability. Sarcopenia is diagnosed when low muscle mass plus decreased muscle strength or weaker physical performance are detected (Chen et al., 2020). When decreased muscle strength, low muscle mass and weaker physical performance are all detected, severe sarcopenia will be considered. The participants without any abnormalities in these three indicators were classified as non-sarcopenia. In this study, severe sarcopenia and sarcopenia were combined for statistical analysis (Chen et al., 2020).

Muscle mass was assessed using direct segmental multifrequency bioelectrical impedance analysis (In-Body 770; Bio space Co., Ltd.). The participants were asked to wear light clothing, remove their shoes and socks, and stand over the electrodes on the machine for 3–5 min. The relative skeletal mass index was calculated by dividing the appendicular skeletal muscle mass (kg) by the square of height (m) (Wang et al., 2021).

Handgrip strength (kg) was measured using an adjustable hydraulic hand-held dynamometer (EH101; CAMRY; range 0–90 kg; accuracy 0.1 kg). Participants were tested by trained evaluators with standardized verbal instructions. The dynamometers were calibrated before testing and adjusted for optimal fit for each participant according to instructions on the dynamometer. Participants were instructed to hold the dynamometer beside but not against their body while standing upright with the arm vertical and then grip the dynamometers as hard as they could. Handgrip strength was measured twice for each hand, and the greater recorded value was considered the maximal grip strength (Wang et al., 2021).

Gait speed over a distance of 6 m was measured to assess muscle performance. Participants were directed to wear flat, comfortable walking shoes and walk for 4 m at their regular speed. The gait speed test was performed only once. The trained evaluators recorded the time using a stopwatch in seconds (Wang et al., 2021).

### Statistical analysis

Data were processed and analysed using R version 4.1.3 (University of Science and Technology of China; 2022–03–10). The one-sample Kolmogorov‒Smirnov test was used to test the normality of the distribution of variables. Characteristics of the data are presented as the means ± standard deviations (SD) and frequencies. Differences between the categories of sarcopenia were analysed through the independent two-sample *t*-test and Pearson’s chi-squared test. The association between the characteristic variables and sarcopenia was analysed by binary logistic regression using three separate models: Model 1, univariate logistic regression models; Model 2, adjusted by age and sex; and Model 3, adjusted by Model 2+ income status and spouse status. The threshold of significance was 0.05.

## Results

### Patient demographics and clinical characteristics

**FIGURE 1:**
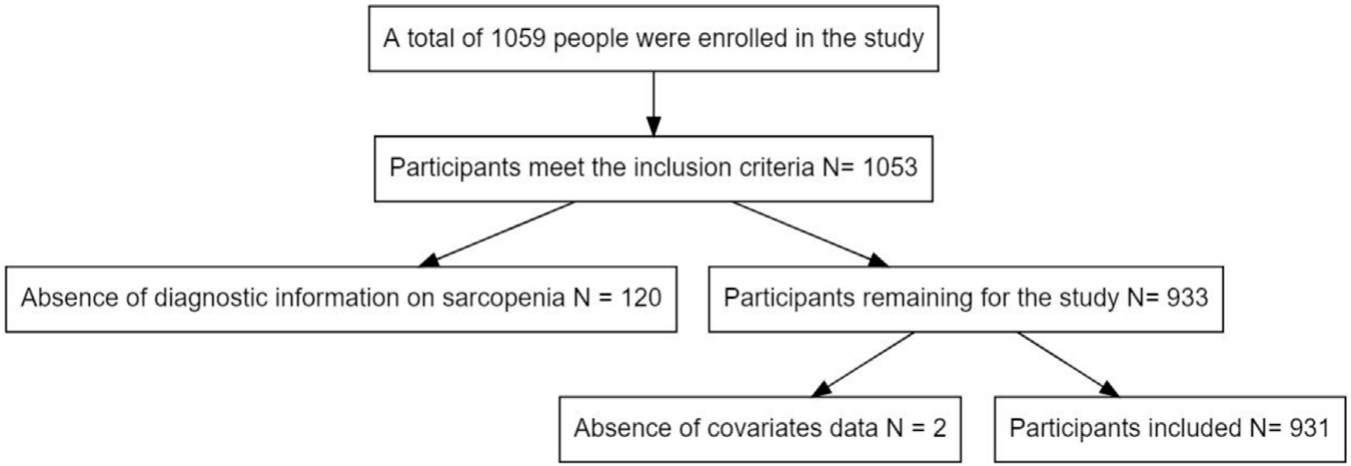
Flow diagram of the participants included in the study.

We recruited 1,059 community seniors in this study. Only 1,053 participants fully met the study inclusion criteria. Among these, 120 participants were excluded from the analysis due to incomplete sarcopenia assessment. Finally, 2 patients were excluded without covariate data. The flow of participants through each stage of selection based on exclusion criteria is shown in Figure 1.

The baseline characteristics of participants by sarcopenia categories are shown in Table 1. There were differences in the basic characteristics of participants between the categories of sarcopenia. The mean age of the participants was 67.9 (±7.6) years. The prevalence of sarcopenia (*n* = 82) was 8.8%. Participants with nutritional risk and malnutrition had a high prevalence of sarcopenia (39%). The results of this study showed that vegetarian participants had a higher risk of sarcopenia (20%). The participants who consumed fruits and nuts daily had a lower risk of sarcopenia (fruit intake every day 8.2%; nut intake every day 5.5%). The participants who had spicy eating habits had a higher risk of sarcopenia (30%).

**TABLE 1:** Sample characteristics stratified by sarcopenia status (N = 931).

Characters	All*	Nonsarcopenia^#^	Sarcopenia^#^	*p*-Value
	N = 931	N = 849	N = 82	
Age, mean (sd)	67.92 (7.62)	67.48 (7.32)	72.5 (9.07)	<0.001
Ages groups, n (%)				<0.001
50–59	120 (12.9%)	114 (95%)	6 (5%)	
60–69	450 (48.3%)	424 (94.2%)	26 (5.8%)	
70–79	281 (30.2%)	252 (89.7%)	29 (10.3%)	
80+	80 (8.6%)	59 (73.8%)	21 (26.2%)	
Sex, n (%)				0.794
Male	244 (26.2%)	224 (91.8%)	20 (8.2%)	
Female	687 (73.8%)	625 (91%)	62 (9%)	
Income status, n (%)				0.097^f^
≤30,000 RMB	39 (4.2%)	34 (87.2%)	5 (12.8%)	
30,000–80,000 RMB	330 (35.4%)	295 (89.4%)	35 (10.6%)	
80,000–200,000 RMB	544 (58.4%)	505 (92.8%)	39 (7.2%)	
20-50w	18 (1.9%)	15 (83.3%)	3 (16.7%)	
Spouse status, n (%)				0.24
Without spouse	197 (21.2%)	175 (88.8%)	22 (11.2%)	
With spouse	734 (78.8%)	674 (91.8%)	60 (8.2%)	
MNA-Nutrition score, mean (sd)	13.44 (0.95)	13.52 (0.87)	12.57 (1.26)	<0.001
Nutritional status, n (%)				<0.001
Normal	890 (95.6%)	824 (92.6%)	66 (7.4%)	
Nutritional risk or malnutrition	41 (4.4%)	25 (61%)	16 (39%)	
Dietary structure, n (%)				0.1^f^
Balanced diet	885 (95.1%)	810 (91.5%)	75 (8.5%)	
Meat diet	16 (1.7%)	15 (93.8%)	1 (6.2%)	
Vegetable diet	30 (3.2%)	24 (80%)	6 (20%)	
Light eating habits, n (%)				0.374
No	83 (8.9%)	73 (88%)	10 (12%)	
Yes	848 (91.1%)	776 (91.5%)	72 (8.5%)	
Salty eating habits, n (%)				0.767
No	843 (90.5%)	770 (91.3%)	73 (8.7%)	
Yes	88 (9.5%)	79 (89.8%)	9 (10.2%)	
Sweet eating habits, n (%)				0.433^f^
No	909 (97.6%)	830 (91.3%)	79 (8.7%)	
Yes	22 (2.4%)	19 (86.4%)	3 (13.6%)	
Spicy eating habits, n (%)				0.05^f^
No	921 (98.9%)	842 (91.4%)	79 (8.6%)	
Yes	10 (1.1%)	7 (70%)	3 (30%)	
Fruit intake, n (%)				0.048
Not everyday	77 (8.3%)	65 (84.4%)	12 (15.6%)	
Everyday	854 (91.7%)	784 (91.8%)	70 (8.2%)	
Vegetable intake, n (%)				1^f^
Not everyday	15 (1.6%)	14 (93.3%)	1 (6.7%)	
Everyday	916 (98.4%)	835 (91.2%)	81 (8.8%)	
Meat intake, n (%)				0.563
Rarely or never	43 (4.6%)	38 (88.4%)	5 (11.6%)	
Weekly or monthly	296 (31.8%)	267 (90.2%)	29 (9.8%)	
Everyday	592 (63.6%)	544 (91.9%)	48 (8.1%)	
Fish intake, n (%)				0.629
Rarely or never	82 (8.8%)	75 (91.5%)	7 (8.5%)	
Weekly or monthly	727 (78.1%)	660 (90.8%)	67 (9.2%)	
Everyday	122 (13.1%)	114 (93.4%)	8 (6.6%)	
Egg intake, n (%)				0.308^f^
Rarely or never	19 (2%)	16 (84.2%)	3 (15.8%)	
Weekly or monthly	211 (22.7%)	190 (90%)	21 (10%)	
Everyday	701 (75.3%)	643 (91.7%)	58 (8.3%)	
Bean intake, n (%)				0.119
Rarely or never	138 (14.8%)	127 (92%)	11 (8%)	
Weekly or monthly	566 (60.8%)	508 (89.8%)	58 (10.2%)	
Everyday	227 (24.4%)	214 (94.3%)	13 (5.7%)	
Pickle intake, n (%)				0.088
Rarely or never	627 (67.3%)	564 (90%)	63 (10%)	
Weekly or monthly	217 (23.3%)	201 (92.6%)	16 (7.4%)	
Everyday	87 (9.3%)	84 (96.6%)	3 (3.4%)	
Carbohydrate intake, n (%)				0.702
Rarely or never	618 (66.4%)	567 (91.7%)	51 (8.3%)	
Weekly or monthly	263 (28.2%)	237 (90.1%)	26 (9.9%)	
Everyday	50 (5.4%)	45 (90%)	5 (10%)	
Garlic intake, n (%)				0.229
Rarely or never	229 (24.6%)	205 (89.5%)	24 (10.5%)	
Weekly or monthly	474 (50.9%)	430 (90.7%)	44 (9.3%)	
Everyday	228 (24.5%)	214 (93.9%)	14 (6.1%)	
Dairy intake, n (%)				0.075
Rarely or never	93 (10%)	79 (84.9%)	14 (15.1%)	
Weekly or monthly	205 (22%)	187 (91.2%)	18 (8.8%)	
Everyday	633 (68%)	583 (92.1%)	50 (7.9%)	
Nut intake, n (%)				0.01
Rarely or never	135 (14.5%)	118 (87.4%)	17 (12.6%)	
Weekly or monthly	415 (44.6%)	371 (89.4%)	44 (10.6%)	
Everyday	381 (40.9%)	360 (94.5%)	21 (5.5%)	
Thallophyte intake, n (%)				0.069
Rarely or never	162 (17.4%)	150 (92.6%)	12 (7.4%)	
Weekly or monthly	564 (60.6%)	505 (89.5%)	59 (10.5%)	
Everyday	205 (22%)	194 (94.6%)	11 (5.4%)	
Vitamin intake, n (%)				0.36
Rarely or never	589 (63.3%)	543 (92.2%)	46 (7.8%)	
Weekly or monthly	110 (11.8%)	99 (90%)	11 (10%)	
Everyday	232 (24.9%)	207 (89.2%)	25 (10.8%)	

^f^ *p* values calculated using exact tests.

* This column counts column percentages.

# This column counts the percentage of rows.

As demonstrated in Table 2, the results from model 1 indicate a significant association between sarcopenia and age, nutrition status, dietary structure, spicy eating habits, fruit intake, dairy intake and nut intake. Furthermore, there was a significant association between sarcopenia and nutrition status, spicy eating habits, fruit intake, pickle intake, dairy intake and nut intake independent of age and sex in model 2. Likewise, the association between cognitive status and sarcopenia was significant when adjusted for age, sex, income status and spouse status, and the association between sarcopenia and fruit intake became nonsignificant in model 3. Variables used to assess sarcopenia stratified by sarcopenia status showed in Table 3.

**TABLE 2:** Factors associated with sarcopenia after adjusting for different factors.

Characters	Model1: OR (95%CI)	Model2: OR (95%CI)	Model3: OR (95%CI)
Sex: Female	1.11 (0.66–1.88)	-	-
Ages groups: 60–69	1.17 (0.47–2.9)	-	-
Ages groups: 70–79	2.19 (0.88–5.41)	-	-
Ages groups: 80+	6.76 (2.59–17.67)	-	-
Income status: 3-8 w	0.81 (0.3–2.2)	0.63 (0.22–1.75)	-
Income status: 8-20 w	0.53 (0.19–1.42)	0.36 (0.13–1)	-
Income status: 20-50 w	1.36 (0.29–6.44)	1.31 (0.27–6.35)	-
Spouse status: With spouse	0.71 (0.42–1.19)	0.98 (0.56–1.71)	-
Nutritional status: Nutritional risk or malnutrition	7.99 (4.07–15.7)	7.38 (3.64–14.98)	7.01 (3.43–14.32)
Dietary structure: Meat diet	0.72 (0.09–5.53)	0.68 (0.08–5.48)	0.6 (0.07–5.01)
Dietary structure: Vegetable diet	2.7 (1.07–6.81)	2.83 (1.09–7.35)	2.57 (0.96–6.87)
Light eating habits: Yes	0.68 (0.34–1.37)	0.63 (0.31–1.31)	0.74 (0.35–1.55)
Salty eating habits: Yes	1.2 (0.58–2.49)	1.2 (0.57–2.56)	1.11 (0.51–2.38)
Sweet eating habits: Yes	1.66 (0.48–5.73)	1.77 (0.48–6.52)	1.64 (0.44–6.07)
Spicy eating habits: Yes	4.57 (1.16–18.01)	7.22 (1.77–29.46)	5.81 (1.35–24.93)
Fruit intake: Everyday	0.48 (0.25–0.94)	0.46 (0.23–0.91)	0.52 (0.26–1.05)
Vegetable intake: Everyday	1.36 (0.18–10.46)	1.35 (0.17–10.81)	1.42 (0.18–11.29)
Meat intake: Weekly or monthly	0.83 (0.3–2.26)	0.79 (0.28–2.23)	0.88 (0.3–2.58)
Meat intake: Everyday	0.67 (0.25–1.78)	0.72 (0.27–1.97)	0.78 (0.28–2.2)
Fish intake: Weekly or monthly	1.09 (0.48–2.46)	1.11 (0.48–2.55)	1.16 (0.5–2.7)
Fish intake: Everyday	0.75 (0.26–2.16)	0.75 (0.25–2.19)	0.75 (0.25–2.23)
Egg intake: Weekly or monthly	0.59 (0.16–2.19)	0.62 (0.16–2.45)	0.81 (0.19–3.38)
Egg intake: Everyday	0.48 (0.14–1.7)	0.56 (0.15–2.09)	0.67 (0.17–2.64)
Bean intake: Weekly or monthly	1.32 (0.67–2.58)	1.68 (0.83–3.38)	1.6 (0.78–3.28)
Bean intake: Everyday	0.7 (0.31–1.61)	0.83 (0.35–1.94)	0.74 (0.31–1.77)
Pickle intake: Weekly or monthly	0.71 (0.4–1.26)	0.63 (0.35–1.14)	0.62 (0.34–1.13)
Pickle intake: Everyday	0.32 (0.1–1.04)	0.3 (0.09–0.99)	0.26 (0.08–0.88)
Carbohydrate intake: Weekly or monthly	1.22 (0.74–2)	1.23 (0.74–2.05)	1.26 (0.75–2.1)
Carbohydrate intake: Everyday	1.24 (0.47–3.25)	1.46 (0.54–3.9)	1.26 (0.46–3.47)
Garlic intake: Weekly or monthly	0.87 (0.52–1.48)	0.9 (0.53–1.55)	0.87 (0.5–1.49)
Garlic intake: Everyday	0.56 (0.28–1.11)	0.63 (0.31–1.27)	0.53 (0.26–1.09)
Dairy intake: Weekly or monthly	0.54 (0.26–1.15)	0.42 (0.19–0.93)	0.49 (0.22–1.08)
Dairy intake: Everyday	0.48 (0.26–0.92)	0.36 (0.18–0.7)	0.38 (0.19–0.75)
Nut intake: Weekly or monthly	0.82 (0.45–1.5)	0.77 (0.42–1.43)	0.83 (0.45–1.56)
Nut intake: Everyday	0.4 (0.21–0.79)	0.41 (0.21–0.82)	0.4 (0.2–0.8)
Thallophyte intake: Weekly or monthly	1.46 (0.76–2.79)	1.4 (0.72–2.7)	1.31 (0.67–2.56)
Thallophyte intake: Everyday	0.71 (0.3–1.65)	0.76 (0.32–1.79)	0.68 (0.28–1.62)
Vitamin intake: Weekly or monthly	1.31 (0.66–2.62)	1.16 (0.57–2.36)	1.18 (0.58–2.43)
Vitamin intake: Everyday	1.43 (0.85–2.38)	1.37 (0.81–2.31)	1.37 (0.81–2.34)

**TABLE 3:** Variables used to assess sarcopenia stratified by sarcopenia status (N = 931).

Characters	All	Nonsarcopenia	Sarcopenia	*p*-Value
	(N = 931)	(N = 849)	(N = 82)	
Grip strength				
Mean (SD)	26.9 (8.60)	27.2 (8.38)	23.3 (9.99)	< 0.001^*^
Median [Q25, Q75]	25.1 [21.25–31]	25.5 [21.5–31.6]	21.85 [17.8–24.9]	< 0.001^#^
6 min walking test				
Mean (SD)	1.01 (0.754)	1.03 (0.780)	0.707 (0.245)	< 0.001^*^
Median [Q25, Q75]	0.857 [0.6–1.2]	0.857 [0.667–1.2]	0.667 [0.545–0.857]	< 0.001^#^
SPPB				
Mean (SD)	10.6 (1.64)	10.7 (1.54)	9.41 (2.06)	< 0.001^*^
Median [Q25, Q75]	11 [10–12]	11 [10–12]	10 [8–11]	< 0.001^#^
SMI				
Mean (SD)	6.83 (1.15)	6.95 (1.12)	5.61 (0.554)	< 0.001^*^
Median [Q25, Q75]	6.7 [6.1–7.4]	6.8 [6.3–7.4]	5.5 [5.3–5.6]	< 0.001^#^

* Two Sample *t*-test.

# Wilcoxon rank sum test.

**FIGURE 2:**
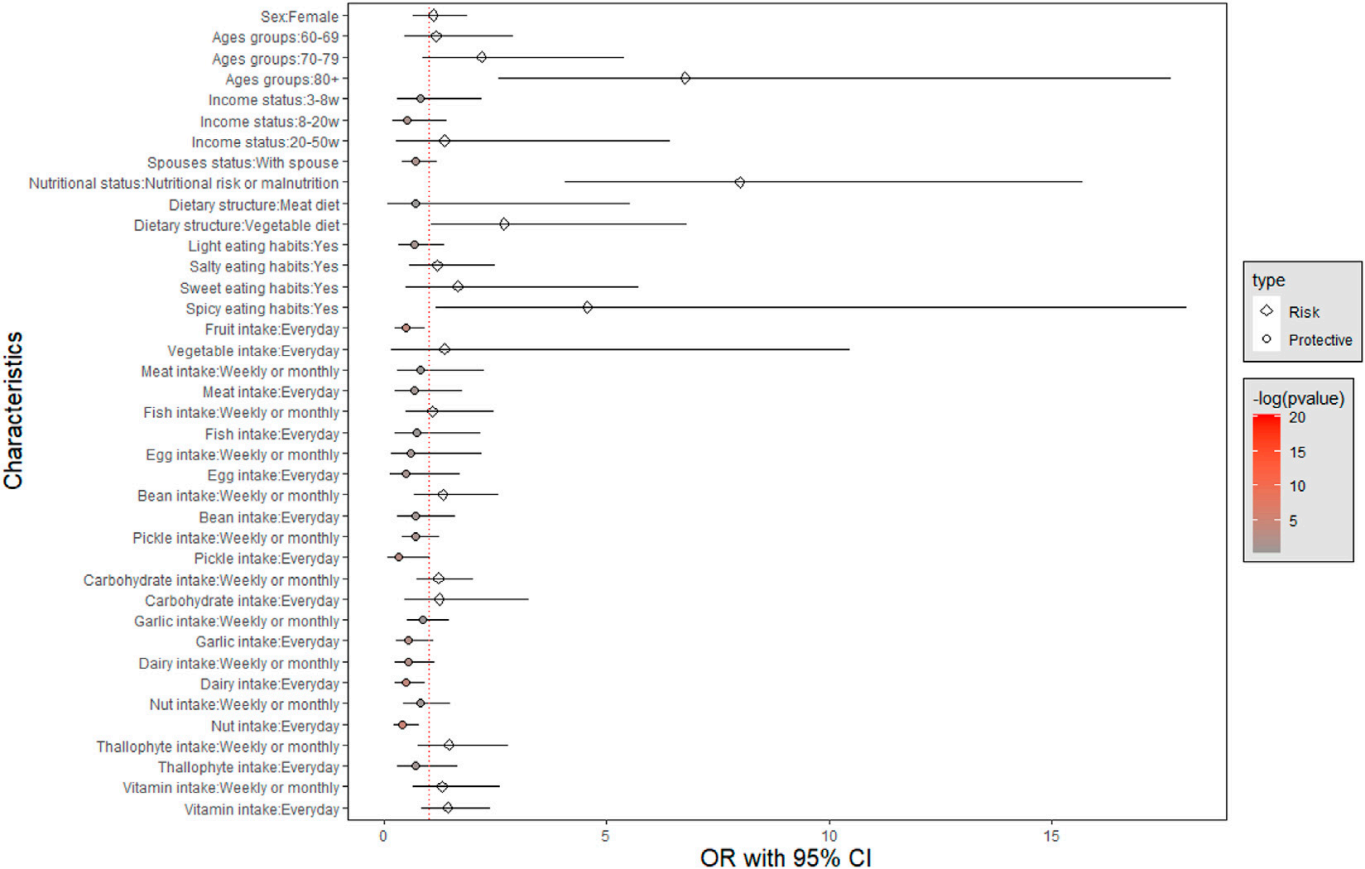
Factors associated with sarcopenia in univariate analysis.

**FIGURE 3:**
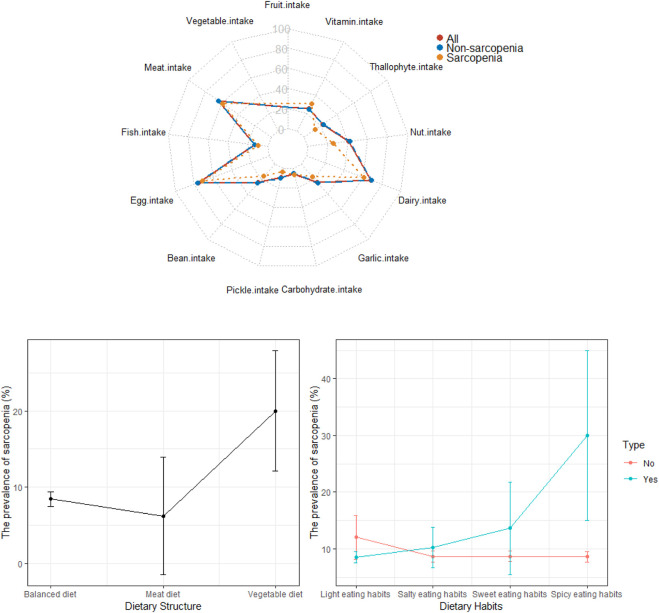
Association of dietary patterns and sarcopenia.

As shown in Figure 2 and Figure 3, age, nutrition status, dietary structure, spicy eating habits, fruit intake, dairy intake and nut intake were associated with sarcopenia. Nutritional risk or malnutrition, vegetable diet, advanced age and spicy eating habits were risk factors for sarcopenia. Daily fruit consumption, daily dairy consumption and daily nut consumption were protective factors against sarcopenia.

## Discussion

According to the updated diagnostic criteria of the AWGS 2019, we found that the overall prevalence of sarcopenia among people aged 50 years or older was 8.8% and increased with age (5% in the 50–59 years group; 5.8% in the 60–69 years group; 10.3% in the 70–79 years group and 26.2% in the ≥80 years group). We identified some nutritional factors associated with sarcopenia, and some interesting results have been found that certain eating habits were associated with sarcopenia.

### Prevalence of sarcopenia

The prevalence of sarcopenia also varies greatly according to the diagnostic criteria of sarcopenia and in different populations (Cruz-Jentoft et al., 2014). According to the diagnostic criteria developed by the AWGS, the prevalence of sarcopenia is approximately 18% (95% CI: 14%–23%) using dual X-ray absorptiometry (DXA) and 14% (95% CI: 11%–16%) using BIA (Petermann-Rocha et al., 2022). A Chinese study involving 6,172 community-dwelling older adults aged ≥60 years showed that the prevalence of sarcopenia is 13.5% in urban areas and 30.9% in rural areas. Sarcopenia also becomes more common in individuals aged 60 years (Wu et al., 2021). The prevalence of sarcopenia in communities aged over 50 in Western China was 19.31% (Liu et al., 2020) and 2.61%–9.72% among those aged over 60 in Eastern China (Huang et al., 2021). In existing studies, the estimated prevalence of sarcopenia varies considerably due to the different diagnostic criteria used, differences in the methods used to measure muscle mass, differences in the cut-off points applied, and heterogeneous study populations (Petermann-Rocha et al., 2022). These factors could all contribute to the large amount of heterogeneity found in the studies. Meanwhile, few studies have reported prevalence in individuals younger than 60 years. Therefore, it is particularly important to study the factors related to sarcopenia in this sample population.

### Sociodemographic factors related to sarcopenia

The sociological reasons that influence sarcopenia are known to be diverse, including aging itself, sex, income, lifestyle, etc. (Gao et al., 2021). However, in previous studies, the factors associated with sarcopenia were varied, and sometimes the results have been inconsistent and controversial (Su et al., 2019; Yang et al., 2020; Gao et al., 2021). Our study demonstrated that sex, marital status (with or without spouse), and income status were not independent factors associated with sarcopenia. However, the incidence of sarcopenia is higher in older age and people with nutritional risk or malnutrition, which is consistent with previous reports (Sieber, 2019). The prevalence of malnutrition risk has been reported to range from 16% to 73% among community-dwelling older adults in Asia, whereas the prevalence of malnourishment can be as high as 22% (Noe et al., 2020; Norazman et al., 2020; Tan et al., 2021a). Several cross-sectional studies in Asia have linked malnutrition and sarcopenia and suggested early identification of associated risk factors in older adults (Tey et al., 2019; Chew et al., 2021). There is also growing evidence that nutritional status may be a modifiable risk factor for the development of muscle health problems, including sarcopenia (Tey et al., 2019; Bruyere et al., 2022; Chen et al., 2022).

### Correlation among dietary structure, eating habits and sarcopenia

Recently, the analysis of dietary patterns has emerged as a useful tool to elucidate the relationship between diet and sarcopenia. The results of this study showed that vegetarian participants had a higher risk of sarcopenia (20%) than a balanced diet pattern of vegetables and meat (8.5%) and meat diet pattern (6.2%). One possible factor thought to contribute to the relationship between vegetarian diets and a higher risk of sarcopenia is the insufficient protein intake of this dietary pattern. Protein intake was positively related to meat product consumption in elderly individuals (Zhao et al., 2021). Evidence from a systematic review concluded that protein supplementation may improve muscle strength and function through muscle protein synthesis or preventing muscle breakdown (Malafarina et al., 2013). In a large-scale cross-sectional study of an elderly Chinese population, three major dietary patterns were identified: the sweet pattern, vegetable pattern and animal food pattern (Wang et al., 2021). This study demonstrated that a higher vegetable pattern and animal food pattern score was related to a lower prevalence of sarcopenia in elderly adults (Wang et al., 2021). A new systematic review showed that the patters high in vegetables (such as the Mediterranean diet) have been connected to sarcopenia. Mediterranean diet adherence had, in general, a positive role in muscle mass and muscle function, while the results were less clear with regard to muscle strength (Van Elswyk et al., 2022). The different age ranges used in these studies may also have contributed to the different results, and further research is needed on the relationship between the patters high in vegetables and sarcopenia. However, a cross-sectional study identified a ”cereals–tubers–animal oils” pattern, a ”mushrooms–fruits–milk” pattern and an ”animal foods” pattern in community-dwelling older people from three regions of China (Li et al., 2020). The ‘animal food’ pattern showed no significant association with sarcopenia in that study (Li et al., 2020), which indicated that protein and fat might play different roles in the development of sarcopenia. Therefore, more evidence is required to determine the association between dietary patterns and sarcopenia.

Furthermore, in our study, the participants who consumed fruits, nuts and dairy daily had a lower risk of sarcopenia. Oxidative stress plays an important role in the pathogenesis of sarcopenia (Nishikawa et al., 2021). Fruits and nuts provide abundant antioxidants, which may contribute to reduced oxidative stress (Del Rio-Celestino and Font, 2020). Nuts are rich in plant protein, unsaturated fatty acids, phytochemicals, vitamins and minerals; therefore, these nutrients may act synergistically to prevent and manage sarcopenia in older adults (Tan et al., 2021b). To date, there have been no intervention studies on the association of nut consumption and sarcopenia in the open literature. Dairy products are good sources of high-quality protein, mainly in the form of whey or casein (Wilkinson et al., 2007). They require no cooking or minimal preparation, making dairy sources a practical option for seniors to consume adequate protein (Hidayat et al., 2018). Evidence from a systematic review demonstrated that dairy product consumption in older adults may reduce the risk of frailty, particularly high consumption of low-fat milk and yogurt, and may also reduce the risk of sarcopenia by improving skeletal muscle mass by adding nutrient-rich dairy proteins to the habitual diet (Cuesta-Triana et al., 2019). Another systematic review and meta-analysis suggested that dairy proteins, at an amount of 14–40 g/d, can significantly increase appendicular muscle mass in middle-aged and older adults without a significant clinical effect on handgrip strength and leg press (Hanach et al., 2019). However, the incidence of lactose intolerance in Chinese adults is about 70% (CA, 2017). For subjects who are lactose intolerant, we recommend drinking yogurt or lactose-free dairy products to improve the intake of dairy products and prevent the occurrence of sarcopenia. Meanwhile, our research team also found for the first time that the participants who had spicy eating habits had a higher risk of sarcopenia. A possible mechanism was their effect on energy expenditure through the thermic effect of food (Yoshioka et al., 1998). Whether a spicy eating habits diet means higher saturated fatty acid intake and hence sarcopenia risk or other pathogenesis remains to be determined.

As shown in Figure 2, nutritional risk or malnutrition, vegetable diet, advanced age and spicy eating habits were risk factors for sarcopenia; daily fruit, dairy and nut consumption were protective factors against sarcopenia.

### Limitations of this study

There are some limitations that should be considered in the present study. First, the cross-sectional study design leads to the uncertainty of a causal relationship. Second, all participants were from Beijing, the northern capital of this large country. Therefore, due to regional differences in dietary patterns, the conclusions of this study may not be applicable to other populations and countries. Third, resulted from the COVID-19 pandemic, the sample size of this study was not sufficient which affected the group discussion and analysis of related factors. Finally, participant bias in reporting food frequency was a potential limitation, as well as single foods rather than patterns were considered. And this study did not investigate the daily nutrient intake of the subjects or some of these unmeasured factors such as exercise/activity level, acute illness/chronic disease but only analysed the dietary structure, which needs to be further improved in follow-up studies. As a result, other potential dietary patterns for the prevention of sarcopenia might not have been identified in the present study. We cannot rule out the possibility that unmeasured factors might contribute to the association observed.

## Conclusion

In conclusion, the present study found that the overall prevalence of sarcopenia in community-living population was 8.8% and increased with age: 5%, 5.8%, 10.3% and 26.2% in the 50–59, 60–69, 70–79, and ≥80 years groups, respectively. Sex, marital status (with or without spouse), and income status were not independent factors associated with sarcopenia. However, nutritional risk or malnutrition, vegetable diet, advanced age and spicy eating habits were risk factors for sarcopenia; daily fruit, dairy and nut consumption were protective factors against sarcopenia. The results add to the growing body of evidence that nutritional status plays a significant role in the development of sarcopenia, and dietary interventions may be an effective strategy in helping prevent sarcopenia.

## Data Availability

The original contributions presented in the study are included in the article/Supplementary Materials, further inquiries can be directed to the corresponding author.

## Abbreviations

MNA-SF, Mini Nutritional Assessment short-form; AWGS, Asian Working Group of Sarcopenia; SPPB, Short Physical Performance Battery; BIA, bioelectrical impedance analysis; SD, standard deviation; DXA, dual X-ray absorptiometry; CI, confidence interval.
